# Socio-Demographic Determinant Factors for Serum Iron, Copper, Zinc, and Selenium Concentrations Among U.S. Women of Childbearing Age

**DOI:** 10.3390/nu16234243

**Published:** 2024-12-09

**Authors:** Anqi Peng, Peipei Hu, Chutian Shi, Angela Vinturache, Guodong Ding, Yongjun Zhang

**Affiliations:** 1 Department of Pediatrics, The Affiliated Hospital of Yangzhou University, Yangzhou University, Yangzhou 225001, China; paq2021@163.com; 2 Clinical Medical College, Yangzhou University, Yangzhou 225009, China; 3 Department of Pediatrics, Xinhua Hospital, Shanghai Jiao Tong University School of Medicine, Shanghai 200092, China; hupphpp@163.com (P.H.); shichutian@sjtu.edu.cn (C.S.); 4 Department of Obstetrics & Gynecology, University of Alberta, Edmonton, AB T6G 2R3, Canada; angela.vinturache@gmail.com; 5 Department of Neuroscience, University of Lethbridge, Lethbridge, AB T1K 3M4, Canada

**Keywords:** trace elements, socio-demographic, women, childbearing age, NHANES

## Abstract

Background: Trace elements (TEs) are essential nutrients for the human body and have a significant impact on fertility and hormone levels in women of reproductive age, underscoring the importance of understanding sociodemographic variations in their concentrations within this population. Objective: To investigate the socio-demographic factors influencing blood concentrations of four essential TEs, including iron, zinc, copper, and selenium among women of reproductive age. Methods: A cross-sectional analysis of women aged 20–44 years was performed using the National Health and Nutrition Examination Survey, 1999–2018. Serum iron data were analyzed for 9211 women across 10 cycles, while serum copper, zinc, and selenium data were available for 1027 women across 3 cycles. Generalized linear and logistic regressions examined the individual associations of socio-demographic factors, including age, race and ethnicity, education, and poverty index ratio, with iron, zinc, copper, and selenium concentrations treated as continuous and categorical outcomes, respectively. A qualitative heatmap explored the joint associations between the socio-demographic factors and the four essential TEs. Results: Reduced iron concentrations and increased risks of insufficiency occurred in older, Black, low-education, or low-income women. Black women were more likely to have lower zinc and selenium concentrations and an increased risk of zinc insufficiency but higher copper concentrations. The qualitative heatmap found that older, Black, low-education, and low-income women generally had lower concentrations of the four TEs, particularly iron (β = −0.10; *p* < 0.01). Conclusions: Socially disadvantaged women are more likely to present with lower TE concentrations, and these specific population groups should be targeted by replenishment planning by public health initiatives.

## 1. Introduction

Iron, zinc, copper, and selenium as trace elements (TEs) in the human body perform a number of important biological functions and are essential for life and health [1,2]. Globally, approximately 2 billion people are affected by deficiencies in one or more TEs, significantly adding to the global burden of various diseases [3]. Insufficiency in these elements is relatively common among childbearing-aged women (20–44 years) due to their increased demands. The health of childbearing-aged women, as well as their reproductive outcomes, are particularly affected by TE insufficiency, even more than was generally considered previously [4]. However, the true extent of TE insufficiencies worldwide is unknown, especially for women of childbearing age.

Reproductive-aged women are vulnerable to nutritional insufficiency because of physiological changes during the period of pregnancy. Low zinc and selenium concentrations in women of reproductive age were closely associated with prolonged time to pregnancy (TTP) and fertility [5,6]. Additionally, concentrations of trace elements may influence the occurrence of reproductive cancers in women of reproductive age. A meta-analysis and Mendelian randomization study found an association between lower blood zinc levels and higher ovarian cancer risk [7], while higher serum selenium concentrations were linked to cervical cancer and may serve as a protective factor against it [8]. Severe insufficiencies of essential TEs likely increase the risks of maternal complications, stillbirth, birth defects, fetal growth restriction, or long-term low neurobehavioral functions [9,10]. Recent studies identified iron, zinc, copper, and selenium as being involved in neurodevelopment, and they were associated, in some studies, with the incidence of intellectual impairments and neurodevelopmental delay [11]. The nutritional roles, requirements, and metabolism and the quantitative relationship between dietary intakes and health for a number of TEs have been more clearly defined in recent years [12,13], but little is known about the causes of variation between people living within similar environments. Some studies have suggested that socio-demographic characteristics such as age, race and ethnicity, marital status, socio-economic status, educational status, and religious belief can play a key role in the determinants of element status in reproductive-aged women. A better understanding of how TE concentrations vary with socio-demographic characteristics might provide a tool to identify subgroups of the population with an increased risk of TE imbalances or deficiencies and potentially associated diseases.

Our primary objective for this study was to estimate the correlation between socio-demographic determinant factors of reproductive-aged women in the U.S. and four TEs by collecting individual-level biomarker data for TE status from the National Health and Nutrition Examination Survey (NHANES) database. Identifying potential determining factors is critical to enabling the formulation of health policies and personalized medical recommendations for women and newborns.

## 2. Materials and Methods

### 2.1. Study Design and Population

The survey comprised face-to-face interviews at participants’ homes, followed by standardized physical examinations at mobile examination centers, where blood samples were collected. The details of this cohort have been previously described [14,15]. All data collection procedures were reviewed and approved by the National Center for Health Statistics (NCHS) Ethics Review Board, and written informed consent was obtained from all NHANES participants prior to their participation in this study (https://www.cdc.gov/nchs/nhanes/irba98.htm#print [accessed on 28 December 2023]). This study followed the Strengthening the Reporting of Observational Studies in Epidemiology (STROBE) reporting guideline for cohort studies.

**Figure 1:**
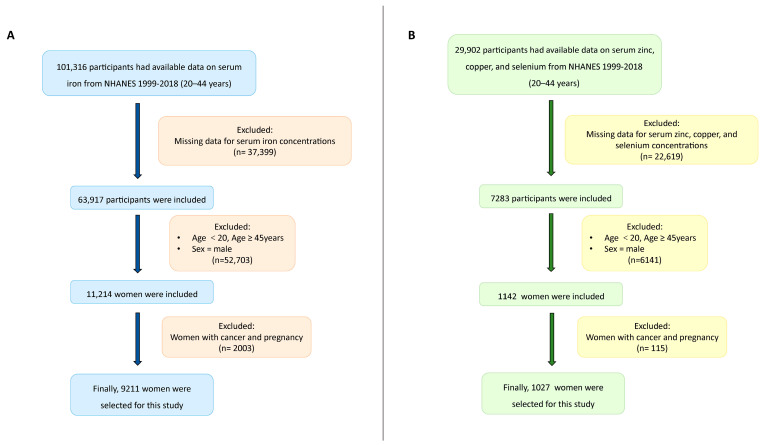
Flowchart of participant selection [serum iron flowchart (**A**); serum zinc, copper, and selenium flowchart (**B**)].

We used data from U.S. women aged 20–44 years who participated in 10 cycles (1999–2000 to 2017–2018) of NHANES. In the 1999–2018 dataset, serum iron was measured in all NHANES participants older than 12 years of age. However, serum zinc, copper, and selenium were examined in a randomly selected one-third subset of NHANES participants older than 6 years of age from 2011 to 2016. A total of 101,316 participants had available data on serum iron and 29,902 women had obtainable data on serum zinc, copper, and selenium. Among them, we excluded the participants who had uncompleted data on the four TEs. Then, we excluded individuals who were >45 years or <20 years or whose sex was male. Further, we removed pregnant women and women with cancer. Finally, 9211 participants were selected for inclusion for serum iron and 1027 were chosen for serum zinc, copper, and selenium (Figure 1A,B).

### 2.2. Socio-Demographic Information

Standardized questionnaires were administered to the participants by trained interviewers who collected demographic information including age, race and ethnicity, education attainment, and poverty status. Age was classified into two groups: 20–34 and 35–44. Race and ethnicity were coded as “Non-Hispanic White”, “Hispanic”, “Non-Hispanic Black”, and “Other Race”. Educational attainment was divided into two categories: high school and below (low education) and some college and above (high education) based on prior studies [16]. Poverty income ratio (PIR) was a measure of socio-economic status, which was categorized into <1.85 (low income) and ≥1.85 (high income) based on prior studies [17].

### 2.3. Outcome Assessment

Serum concentrations of iron, zinc, copper, and selenium were obtained from the NHANES database, where samples were collected at mobile examination centers and analyzed by certified laboratories following standardized protocols. For detailed testing methods, please refer to the Laboratory Methods (https://wwwn.cdc.gov/nchs/nhanes/continuousnhanes/labmethods.aspx?Cycle=2021-2023 [accessed on 28 December 2023]).

We obtained the reference ranges for the included elements based on a literature review [18]. Serum iron (9.0–30.4 μmol/L) and copper (12.6–24.4 μmol/L) levels typically exhibited sex differences, so separate ranges were applied for males and females. In contrast, serum zinc (10.7–22.9 μmol/L) and selenium (46.0–143.0 μg/L) concentrations generally showed no significant variation by sex. Therefore, a single adult reference range was used for these elements.

### 2.4. Statistical Analysis

We conducted all analyses using appropriate methods for structured survey data, incorporating sample weights, strata, and primary sampling units to produce nationally representative estimates. Population demographics were evaluated using weighted descriptive statistics, including proportions for categorical variables. The chi-squared test or Mann–Whitney U test was used to compare the categorical variables as needed, with the Mann–Whitney U test specifically applied to compare trace element concentrations. The distribution of 4 TE concentrations was presented by the selected percentiles. Due to the skewed distribution of 4 TEs, a log_10_ transformation for continuous variables was performed prior to using this variable in the regression models.

The associations of 4 serum TE concentrations as continuous variables with individual socio-demographic factors in women were first analyzed by generalized linear regression models. The concentrations of these TEs were classified as binary variables (sufficiency vs. insufficiency) according to standard reference ranges and analyzed using logistic regression models. Covariates, including sex, age group, race and ethnicity, educational attainment, and PIR, were selected for adjustment based on prior literature and their established influence on both exposure (TE concentrations/insufficiency) and outcomes (socio-demographic factors). To further evaluate the potential joint effects of these significant socio-demographic data, a heatmap was employed to visually explore the joint associations between socio-demographic factors and TE concentrations and insufficiency rates by generalizing the linear and logistic regression models. Heatmaps are particularly effective for presenting complex interactions, offering an intuitive overview of correlations. Red indicates negative correlations and risk factors, while blue represents positive correlations and protective factors, facilitating the interpretation of patterns across multiple dimensions. All statistical analyses were performed using R studio (R version 4.3.2, packages “survey”, “dplyr”, and “gtsummary”) and significance was determined at *p* < 0.05 (two-tailed).

## 3. Results

A total of 9211 participants in the analytic sample had serum iron data available, while 1027 participants had serum zinc, copper, and selenium data. The sample included in our study, apart from educational attainment, did not significantly differ from the general population in other demographic groups, making the enrolled participants representative of the population. Serum iron and zinc levels in reproductive-age women were generally lower than what was observed in the overall female population. Specifically, non-Hispanic White individuals, as well as those with higher educational attainment and higher income levels, were the most prevalent groups in our study. The median (IQR) concentrations of the serum iron, zinc, copper, and selenium were 13.3 (9.3, 18.1) μmol/L, 11.8 (10.4, 13.2) μmol/L, 19.8 (16.9, 23.6) μmol/L, and 123.5 (115.0, 134.8) μg/L, respectively (Table 1).

**Figure 2:**
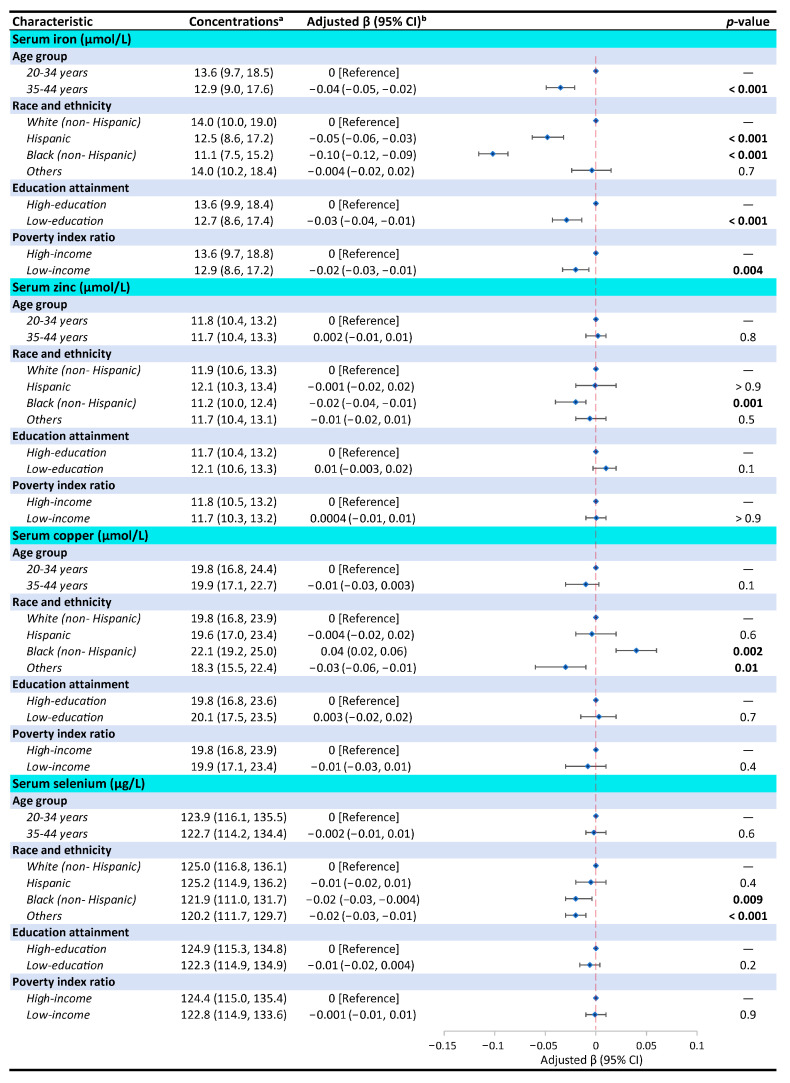
Weighted median (P25, P75) and adjusted β [95% confidence interval (CI)] of each element’s concentrations by socio-demographic subgroups using generalized linear regression model among women aged 20 to 44 years from NHANES. ^a^ The concentrations of each element were not subjected to log_10_ transformation. ^b^ Adjusted for confounders including sex, age group, race and ethnicity, education attainment, and poverty index ratio.

As shown in Figure 2, individual associations of socio-demographic factors, including age, race and ethnicity, education, and PIR, with serum iron, zinc, copper, and selenium concentrations were performed by generalized linear regression analysis. Reduced iron concentrations occurred in older [beta (β) = −0.04; 95% confidence interval (95% CI): −0.05, −0.02; *p* < 0.001], non-Hispanic Black (β = −0.10; 95% CI: −0.12, −0.09; *p* < 0.001), low-education (β = −0.03; 95% CI: −0.04, −0.01; *p* < 0.001), and low-income (β = −0.02; 95% CI: −0.03, −0.01; *p* = 0.004) women. For the subgroup with serum zinc, copper, and selenium, we only found a significant difference related to race and ethnicity. Black women had lower serum zinc (β = −0.02; 95% CI: −0.04, −0.01; *p* = 0.001) and selenium (β = −0.02; 95% CI: −0.03, −0.004; *p* = 0.009), but serum copper (β = 0.04; 95% CI: 0.02, 0.06; *p* = 0.002) was higher.

**Figure 3:**
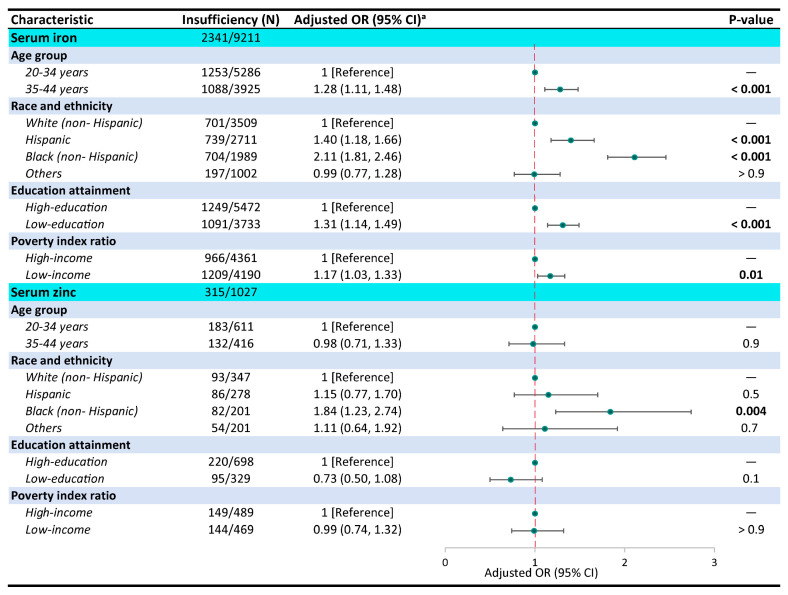
Unweighted sample sizes and adjusted odds ratio (OR) (95% CI) of each element’s insufficiency by socio-demographic subgroups using logistic regression model among women aged 20 to 44 years from NHANES. ^a^ Adjusted for confounders including sex, age group, race and ethnicity, education attainment, and poverty index ratio.

Due to serum copper and selenium concentrations among women almost meeting the reference ranges, these two elements were not included in further statistical analyses. Figure 3 illustrates the associations between individual socio-demographic factors and serum iron and zinc insufficiency rates according to the logistic regression model. Older [odds ratio (OR) = 1.28; 95% CI: 1.11, 1.48; *p* < 0.001], non-Hispanic Black (OR = 2.11; 95% CI: 1.81, 2.46; *p* < 0.001), low-education (OR = 1.31; 95% CI: 1.14, 1.49; *p* < 0.001), and low-income (OR = 1.17; 95% CI: 1.03, 1.33; *p* = 0.01) women had increased odds of serum iron insufficiency. Only non-Hispanic Black women were associated with rising odds of serum zinc insufficiency (OR = 1.84; 95% CI: 1.23, 2.74; *p* = 0.004).

**Figure 4:**
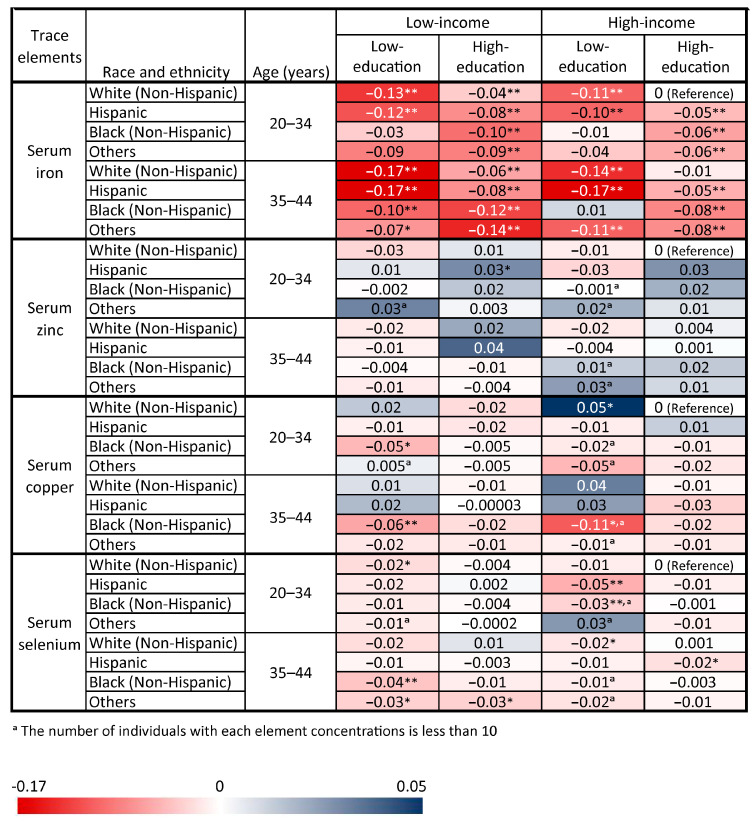
Beta coefficients (β) of the joint associations of these significant socio-demographic factors with each element among women aged 20–44 years from NHANES. * *p* < 0.05, ** *p* < 0.01.

**Figure 5:**
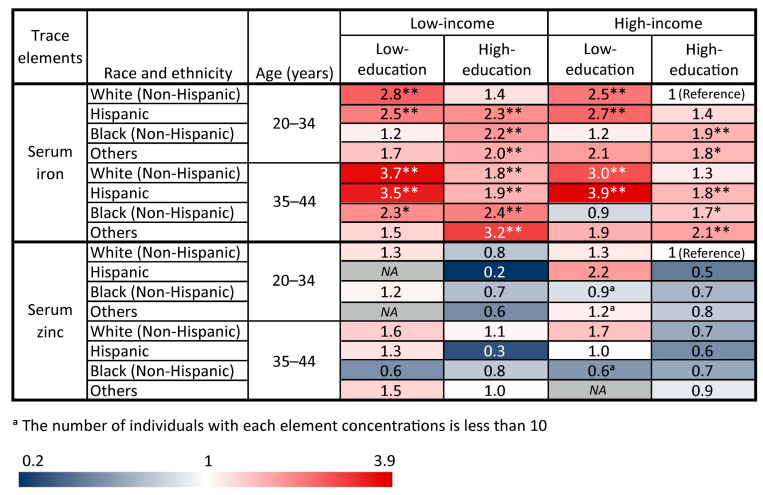
Odds ratio (OR) of the joint associations of these significant socio-demographic factors with serum iron and zinc insufficiency rates among women aged 20–44 years from NHANES. * *p* < 0.05, ** *p* < 0.01. *NA*: No positive samples were present in this group.

Figure 4 and Figure 5 present a heat map illustrating the joint associations of these significant socio-demographic factors including age, race and ethnicity, education attainment, and PIR with TE concentrations (iron, zinc, copper, and selenium) and insufficiency rates (iron and zinc) of women (20–44 years). Given the high prevalence of trace elements observed in non-Hispanic White women aged 20–34 years with a high education and high income, this demographic was selected as the reference group for this study. Figure 2 and Figure 3 reveal that Black women aged 35 to 44 years old with a low education and low income were at an increased risk of having lower serum iron concentrations by 1.26 mmol/L (β = −0.10, *p* < 0.01) and a 130% increase in iron insufficiency rates (OR = 2.30, *p* < 0.05). Furthermore, women of childbearing age who were older, Black, low-education, and low-income generally exhibited lower concentrations of four TEs, with serum iron showing an increased insufficiency rate in this group. In addition, sample sizes for each group in the heatmaps are provided in the Supplementary Data (Tables S1 and S2).

## 4. Discussion

This study estimated the individual and joint associations of socio-demographic factors with TE concentrations and insufficiency rates among women of childbearing age in the U.S. Our study demonstrated that lower TE concentrations and higher insufficiency rates are generally observed among older (35–44 years), non-Hispanic Black, low-education, low-income women, particularly for serum iron.

Stratified analyses suggested that age modified the negative association between socio-demographic factors and TE concentrations and insufficiency rates. We found that women aged 35–44 years reported decreased serum iron concentrations and increased iron insufficiency rates. Pre-menopausal women tend to have a poor iron status due to the combination of low dietary intake and bone loss [19,20]. In preclinical research, iron deficiency anemia (IDA) and latent iron deficiency were quite common in premenopausal nonpregnant women [21]. Decreasing serum iron concentrations are a prerequisite for the development of anemia. In our study, older women tended to have lower TE concentrations and higher iron insufficiency rates. Our results reinforce the need for concerted efforts to promote sufficient iron intake in older women for preventive supplementation.

The effect of race and ethnicity on nutrient-based diet quality cannot be discounted. Our study found that Black women tended to have lower TE concentrations and higher insufficiency rates, with the exception of serum copper, which is consistent with existing evidence. Several studies have also reported that Black women, compared to White women, often have lower levels of key micronutrients, including iron, iodine, and zinc, further highlighting disparities in nutrient status across different populations [22,23]. In line with these findings, Costa et al. (2023) outlined a protocol for a systematic review and meta-analysis to evaluate the global, regional, and national prevalence of copper, selenium, and zinc deficiencies in women of childbearing age, which is expected to further elucidate these disparities once the results are available [24]. Black women are often affected by a lack of nutrition or imbalanced diets due to inadequate access to health services. Available evidence demonstrates that multiple-micronutrient-fortified milk can increase concentrations of TEs and reduce the risk of anemia [25]. Several studies have shown that Black women, particularly those from developing countries, primarily consume non-fortified milk, which may lead to micronutrient deficiencies [26,27,28]. Research indicates that Black women’s diets may be imbalanced, with high calorie intake from fats, carbs, and sugars but insufficient folate, vitamins, and other essential elements [29]. Furthermore, Black households are more likely than White households to experience food insecurity, which is a major risk factor for nutritional problems [30]. In contrast, a study of 1990 adults found no link between race and iron levels, but Black adults had lower copper levels [31]. Our study provides additional evidence supporting the racial disparities in micronutrient status, particularly among Black women.

Income and education are two of the largest components of government policy that the U.S. government can systematically address. Notably, the socio-economic status of people has been improving against the backdrop of social progression. Despite development, disparities persist. Consistent with prior studies, we found lower serum iron concentrations and higher insufficiency rates among women with a low education and low income [32]. To our knowledge, people with a high education and high income are more likely to ignore food access and decrease their variety of consumption, especially regarding fruits and vegetables. A study based on the National Diet and Nutrition Survey (N = 231) found that low-income individuals were at a higher risk of micronutrient inadequacy [33]. Another observational study (N = 340) also found that community intervention health education improved women’s knowledge, which could improve the supplementation of iron [34]. However, previous studies were based on small sample sizes, limiting their representativeness and generalizability. In this study, we employed a large representative population (n > 1000) and observed that a high education and high income were inversely correlated with serum iron concentrations and positively associated with insufficiency rates.

Another notable finding of this study that we explored was the association between joint socio-demographic factors and TE concentrations and insufficiency rates. The joint associations were confirmed by the heat map, which was generally consistent with the individual results. With the aid of the heatmap’s intuitive presentation, the effectiveness of the joint analysis based on socio-demographic factors was vividly demonstrated. These findings can inform public health initiatives by identifying populations at a higher risk of TE insufficiencies, particularly those from socioeconomically disadvantaged groups. For instance, targeted nutritional interventions such as supplementation programs, food fortification, or educational campaigns can be developed to address specific deficiencies within these groups. Additionally, policymakers can utilize these insights to allocate resources more effectively, ensuring that marginalized populations receive adequate support. By adopting such measures, this research contributes to a broader understanding of health inequities and supports efforts to mitigate the adverse effects of TE insufficiencies in vulnerable communities.

Our study has several strengths. First, it utilized a large and comprehensive dataset of the most recent nationally representative data to investigate the health status of U.S. reproductive-aged women regarding TE concentrations compared to established guidelines. Notably, the four elements (iron, zinc, copper, and selenium) included in this study collectively contributed to neurodevelopment. Third, our findings investigated comprehensive factors in individual and joint subgroups. Focusing on these factors can inform broader strategies to enhance healthcare effectiveness. Finally, we identified a specific subgroup that included older, Black, low-education, and low-income women. There are important implications for both policy and clinical practice from our study.

Our study also has several limitations in terms of knowledge. Due to changes between NHANES cycles, some TEs, such as serum zinc, copper, and selenium, are relatively new additions, so sample sizes of reproductive-aged women were small compared to those for serum iron. Second, zinc and selenium lacked sex-specific reference ranges. This could potentially have led to inaccurate assessments of the concentrations of those elements among women. However, there were instances in several studies where this was the case. Third, within the joint socio-demographic factor analysis, the limited sample size (<10) in certain subgroups may have compromised the robustness of the results.

## 5. Conclusions

Our results showed a negative relationship between socio-demographic factors and TE concentrations and insufficiency rates. A distinctive subgroup was identified that included older, Black, low-education, and low-income women of reproductive age. We urge all sectors of society, the government, and healthcare institutions to join forces, redouble their efforts, and pay close attention to the micro-nutrition of these special groups of women and work together to safeguard their health rights.

## Figures and Tables

**Table 1:** Unweighted sample sizes and weighted frequencies and median (P25, P75) of TE concentrations among women aged 20 to 44 years from the National Health and Nutrition Examination Survey (NHANES) ^a^.

Characteristic	Study Participants	Overall Women	*p*-Value
Serum iron ^b^			
No.	9211	32,657	
Race and ethnicity ^e^			<0.001
White (non-Hispanic)	3509 (60.9%)	13,009 (67.0%)	
Hispanic	2711 (18.0%)	9573 (14.3%)	
Black (non-Hispanic)	1989 (13.1%)	7183 (11.8%)	
Other	1002 (8.0%)	2892 (7.0%)	
Education attainment ^d^			<0.001
High education	5472 (65.4%)	13,036 (60.0%)	
Low education	3733 (34.7%)	12,271 (40.0%)	
Poverty index ratio ^d^			0.8
High income	4361 (60.9%)	15,268 (64.4%)	
Low income	4190 (39.1%)	14,581 (35.6%)	
Serum iron (μmol/L) ^f^	13.3 (9.3, 18.1)	13.6 (10.0, 17.9)	<0.001
Serum zinc, copper, and selenium ^c^			
No.	1027	3685	
Race and ethnicity ^e^			0.2
White (non-Hispanic)	347 (56.0%)	1246 (63.6%)	
Hispanic	278 (20.2%)	1070 (16.1%)	
Black (non-Hispanic)	201 (13.0%)	795 (11.9%)	
Others	201 (10.9%)	574 (8.5%)	
Education attainment ^d^			<0.001
High education	698 (72.0%)	1533 (67.0%)	
Low education	329 (28.0%)	1080 (33.0%)	
Poverty index ratio ^d^			0.2
High income	489 (59.3%)	1649 (62.4%)	
Low income	469 (40.8%)	1726 (37.6%)	
Serum zinc (μmol/L) ^f^	11.8 (10.4, 13.2)	12.2 (10.8, 13.5)	<0.001
Serum copper (μmol/L) ^f^	19.8 (16.9, 23.6)	19.4 (16.9, 22.6)	0.020
Serum selenium (μg/L) ^f^	123.5 (115.0, 134.8)	125.0 (115.0, 136.2)	0.91

^a^ All estimates, except sample sizes, are weighted. ^b^ The data for serum iron levels include ten cycles from 1999 to 2018. ^c^ The data for serum zinc, copper, and selenium levels include three cycles from 2011 to 2016. ^d^ Available data of 20–44-year-old women had missing values. ^e^ Hispanic includes Mexican American and other Hispanic. Others includes non-Hispanic multiracial ethnicity. ^f^ The concentrations of each element were not subjected to log_10_ transformation.

## Data Availability

The data used in this study are freely available for download by the public at https://wwwn.cdc.gov/nchs/nhanes (accessed on 28 December 2023).

## Supplementary Materials

The following supporting information can be downloaded at https://www.mdpi.com/article/10.3390/nu16234243/s1: Table S1: Unweighted heatmap sample sizes of iron concentrations among childbearing women aged 20 to 44 years; Table S2: Unweighted heatmap sample sizes of zinc, copper, and selenium concentrations among childbearing women aged 20 to 44 years.
