# Supplementary material for: Socio-Demographic Determinant Factors for Serum Iron, Copper, Zinc, and Selenium Concentrations Among U.S. Women of Childbearing Age
Source: Nutrients. 2024 Dec 9;16(23):4243. doi: 10.3390/nu16234243 (PMC11644430; doi:10.3390/nu16234243)
Supplement: Supplementary file 1 [file nutrients-16-04243-s001.zip › nutrients-3340182-supplementary.pdf]

Table S1 Unweighted heatmap sample sizes of iron's concentrations among childbearing women aged 20 to 44 years

Race and ethnicity	Age (years)	Low-income		High-income	
		Low-education	High-education	Low-education	High-education
White (Non-Hispanic)	20-34 years	350	461	234	878
Hispanic		612	261	167	343
Black (Non-Hispanic)		345	331	92	321
Others		60	130	35	293
White (Non-Hispanic)	35-44 years	257	251	198	736
Hispanic		480	146	163	242
Black (Non-Hispanic)		189	171	85	309
Others		66	77	29	235

Table S2 Unweighted heatmap sample sizes of zinc, copper, and selenium's concentrations among childbearing women aged 20 to 44 years

Race and ethnicity	Age (years)	Low-income		High-income	
		Low-education	High-education	Low-education	High-education
White (Non-Hispanic)	20-34 years	30	68	19	91
Hispanic		52	37	20	46
Black (Non-Hispanic)		32	41	8	30
Others		9	28	7	54
White (Non-Hispanic)	35-44 years	18	28	11	68
Hispanic		43	15	14	26
Black (Non-Hispanic)		19	21	4	35
Others		11	17	3	53
